# Supplementary material for: Polymeric Nanoparticles with Embedded Eu(III) Complexes as Molecular Probes for Temperature Sensing
Source: Molecules. 2022 Dec 12;27(24):8813. doi: 10.3390/molecules27248813 (PMC9785794; doi:10.3390/molecules27248813)
Supplement: Supplementary file 1 [file molecules-27-08813-s001.zip › molecules-2058128-supplementary.pdf]

Electronic supporting information for the article

Polymeric nanoparticles with embedded Eu(III) complexes as molecular probes for temperature sensing

Kirill M. Kuznetsov, Vadim A. Baigildin, Anastasia I. Solomatina, Ekaterina E. Galenko, Alexander F. Khlebnikov, Victor V. Sokolov, Sergey P. Tunik*, Julia R. Shakirova*

Synthesis of ligands and complexes

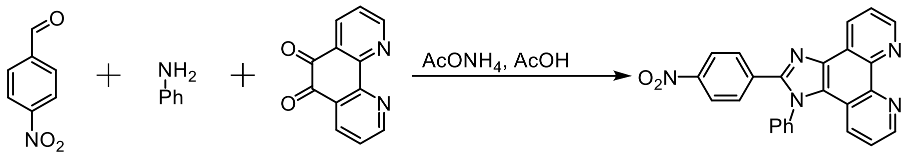

Scheme S1. Scheme of **L3** ligand synthesis.

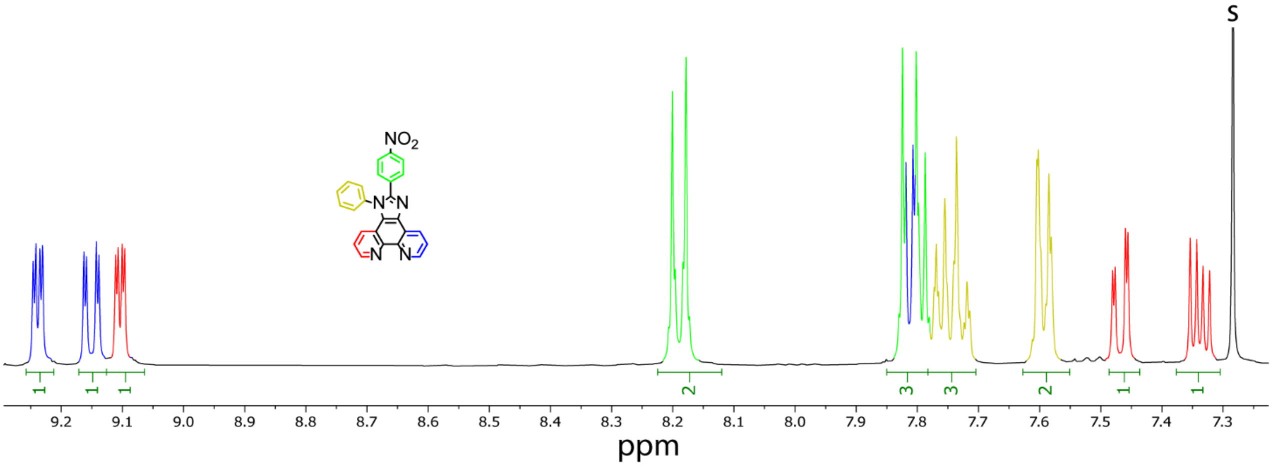

Figure S1. ^{1}H NMR spectrum of ligand $\mathbf{L_3}$ in $CDCl_3$. S – residual solvent peak.

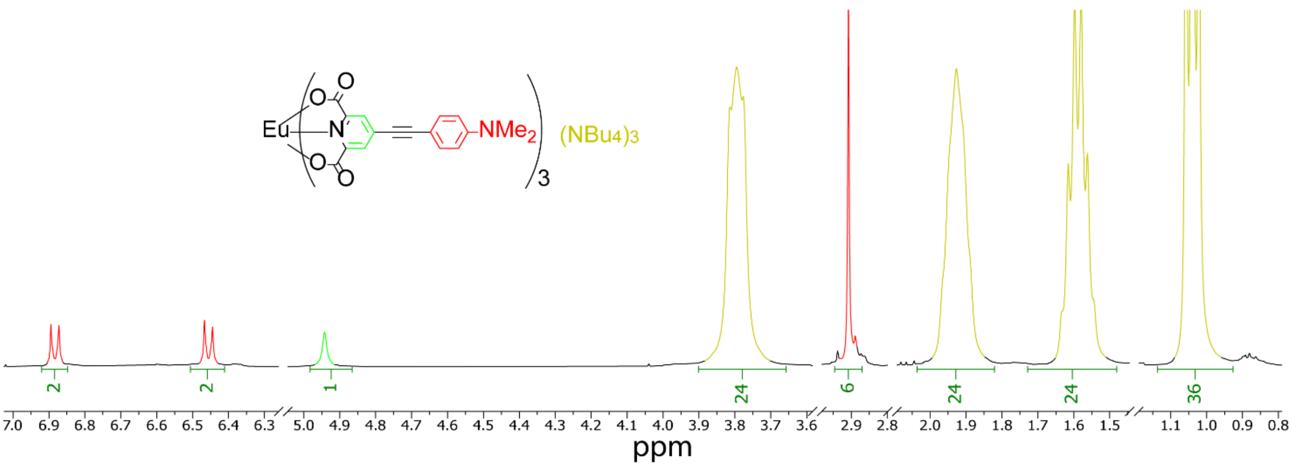

Figure S2. ^{1}H NMR spectrum of complex **Eu1** in $CDCl_3$.

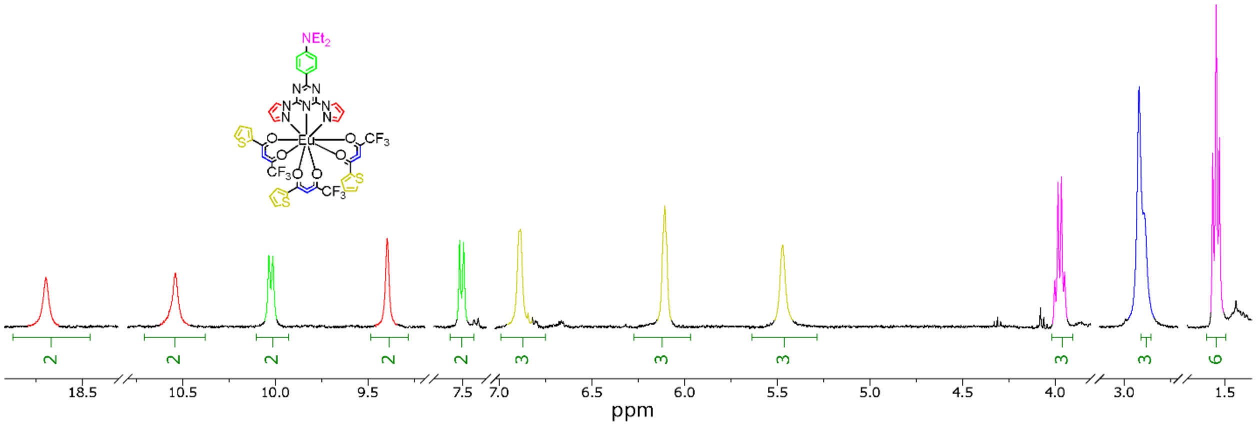

Figure S3. ^{1}H NMR spectrum of complex **Eu2** in DMSO-d_6.

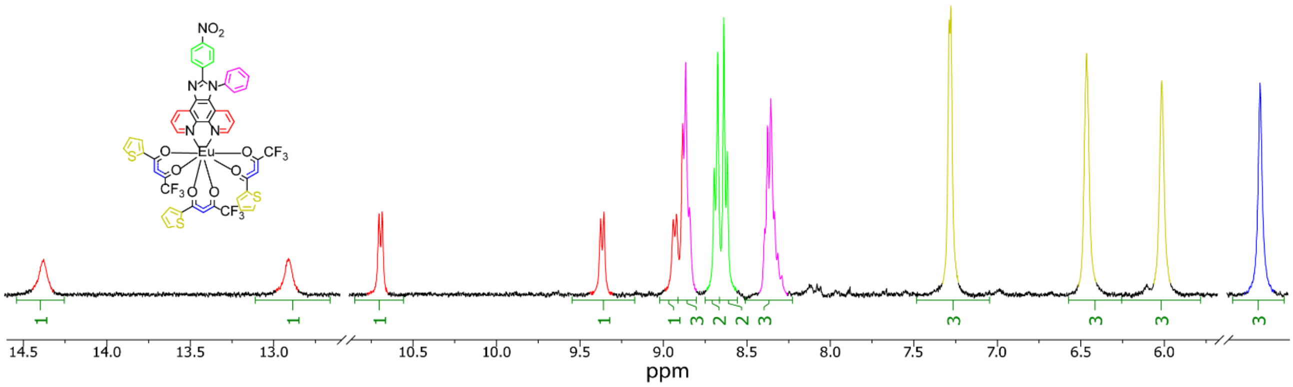

Figure S4. ^{1}H NMR spectrum of complex **Eu3** in Acetone-d_6.

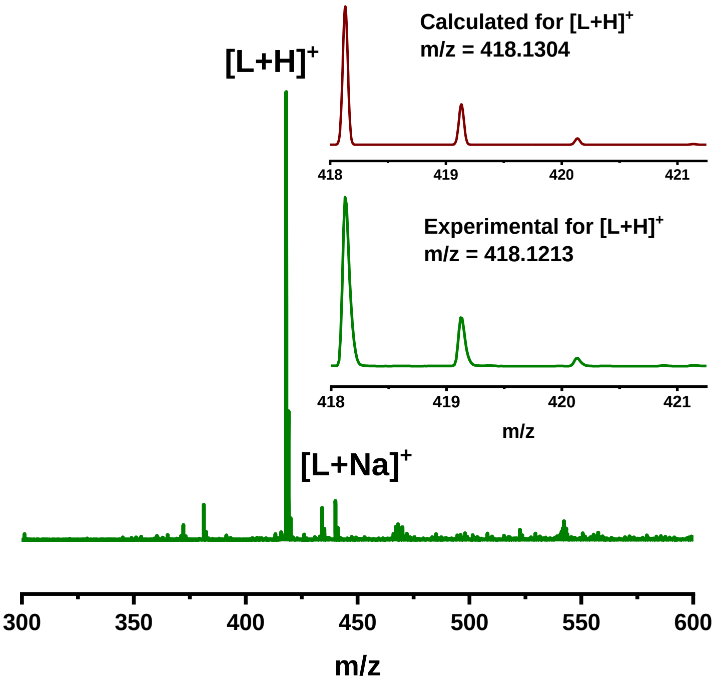

Figure S5. Fragment of ESI$^+$ spectrum of ligand **L3**.

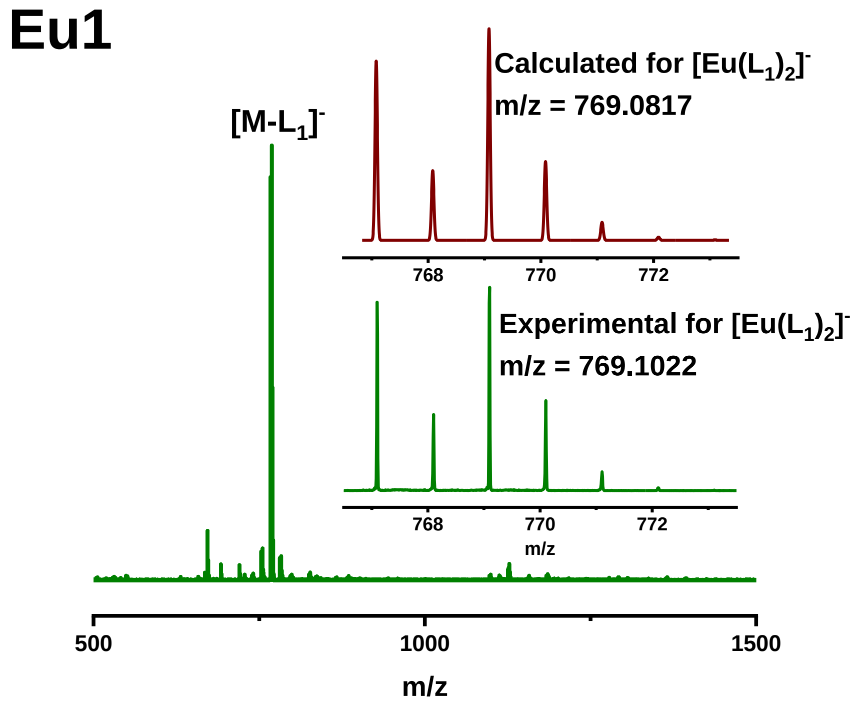

Figure S6. Fragment of ESI$^-$ spectrum of complex **Eu1**.

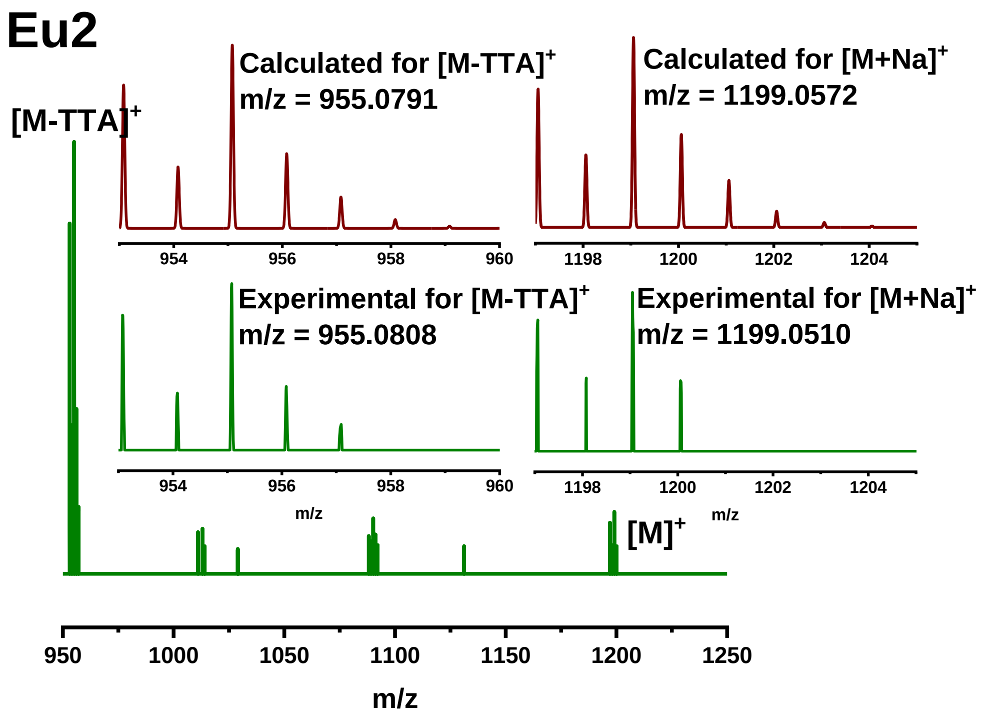

Figure S7. Fragment of ESI$^+$ spectrum of complex **Eu2**.

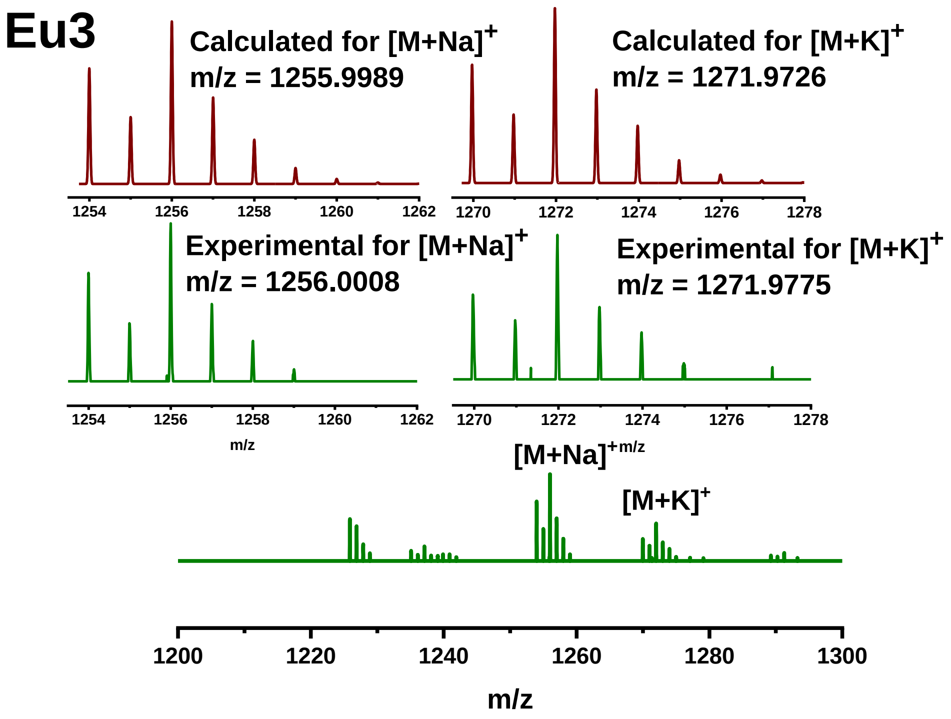

Figure S8. Fragment of ESI$^+$ spectrum of complex **Eu3**.

Photophysical properties of ligands and complexes

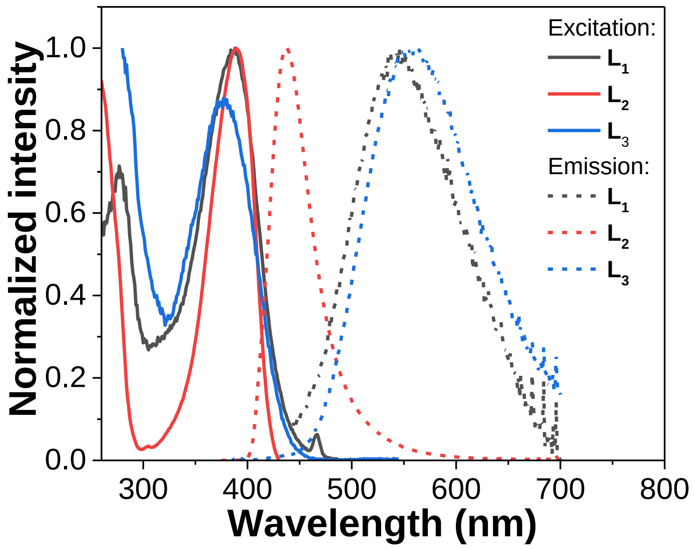

Figure S9. Excitation (solid lines) and emission (dashed lines) spectra of ligands in dichloromethane, 293K.

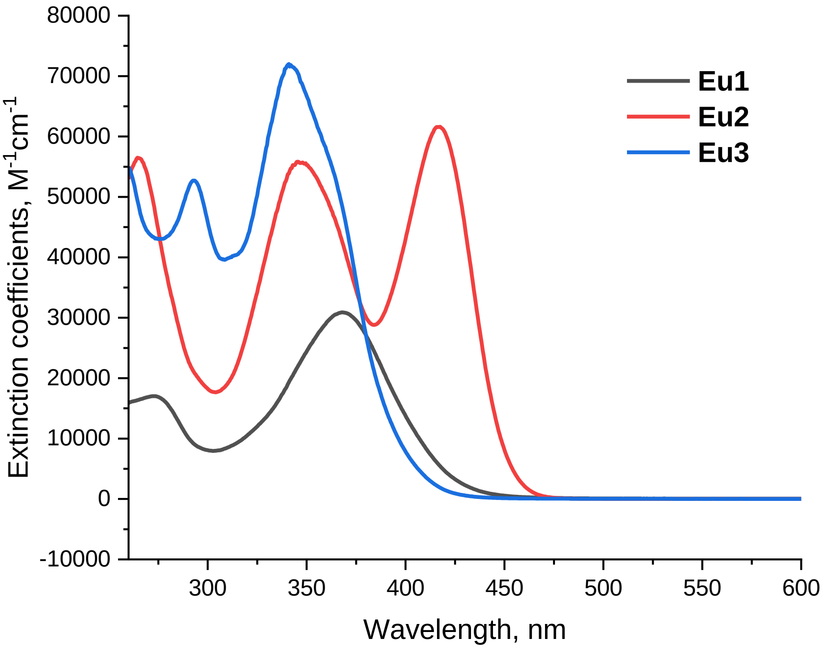

Figure S10. Absorption spectra of **Eu1-Eu3** complexes in dichloromethane, 293K.

Eu1

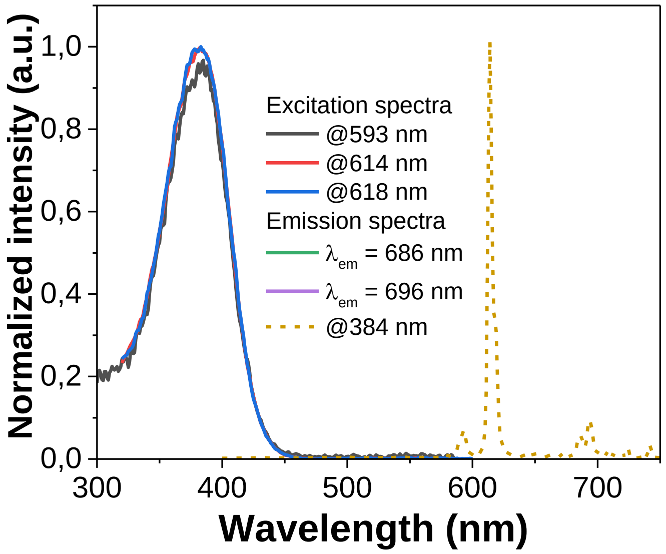

Figure S11. Excitation (solid lines) and emission (dashed lines) spectra of complex **Eu1** in dichloromethane, 293K.

Eu3

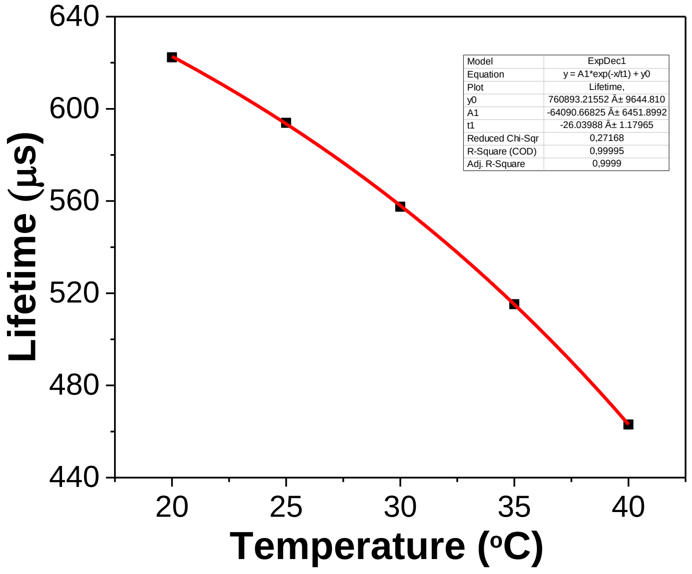

Figure 12. Correlation between lifetimes of excited state and temperature for **Eu3** in dichloromethane solution.

Eu3

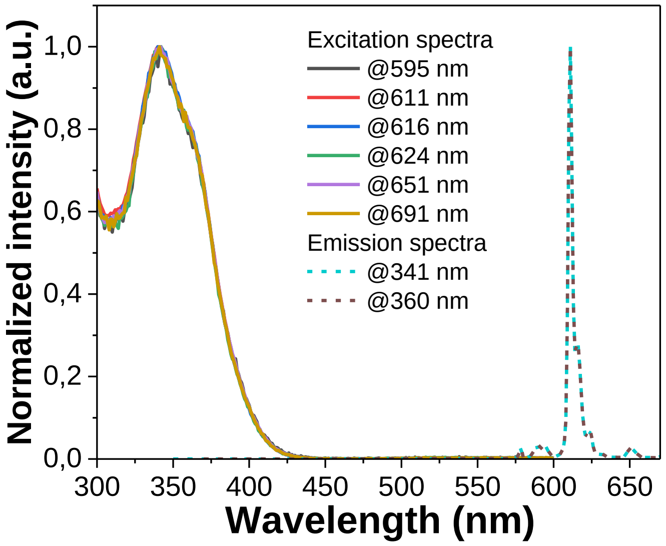

Figure S13. Excitation (solid lines) and emission (dashed lines) spectra of complex **Eu3** in dichloromethane, 293K.

Eu1

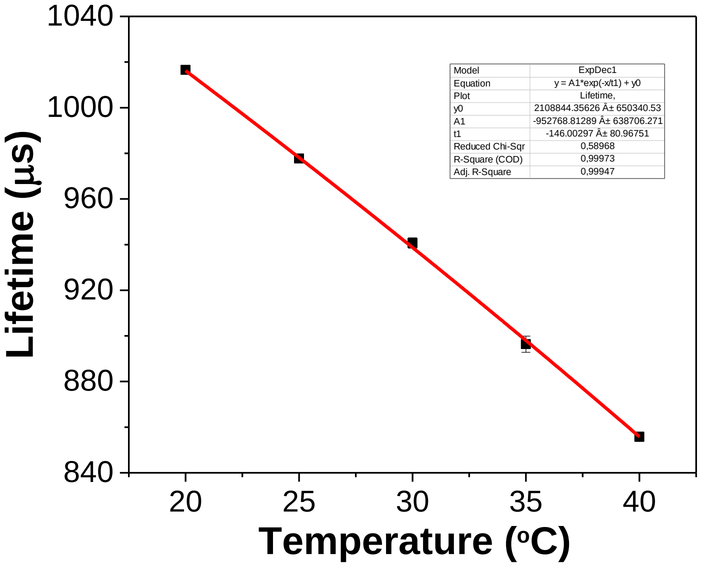

Figure S14. Correlation between lifetimes of excited state and temperature for **Eu1** in dichloromethane solution.

Eu1

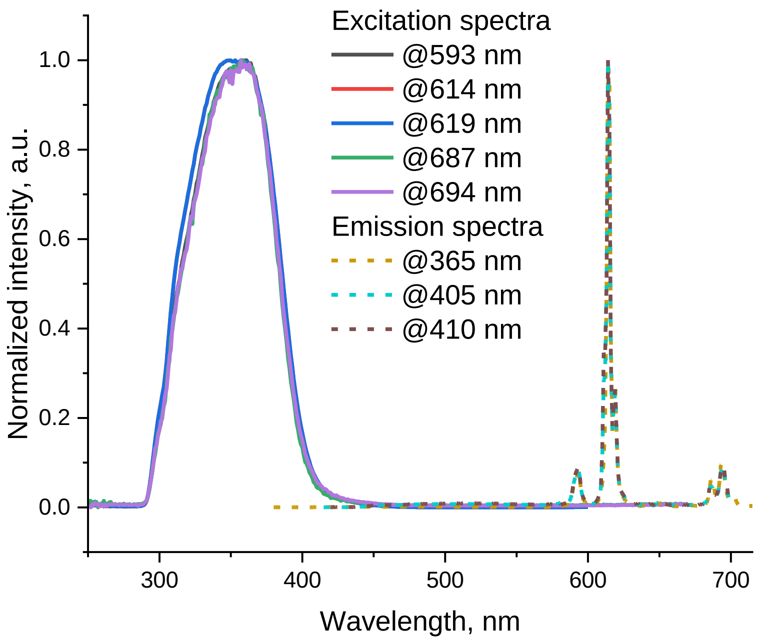

Figure S15. Excitation (solid lines) and emission (dashed lines) spectra of complex **Eu1** in monomers taken with the same amount as for polymerization, 293K.

Eu2

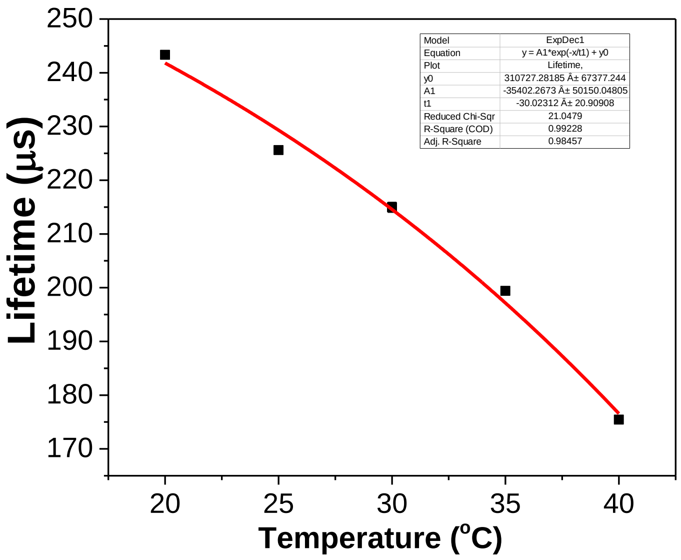

Figure S16. Correlation between lifetimes of excited state and temperature for **Eu2** in dichloromethane solution.

Eu2

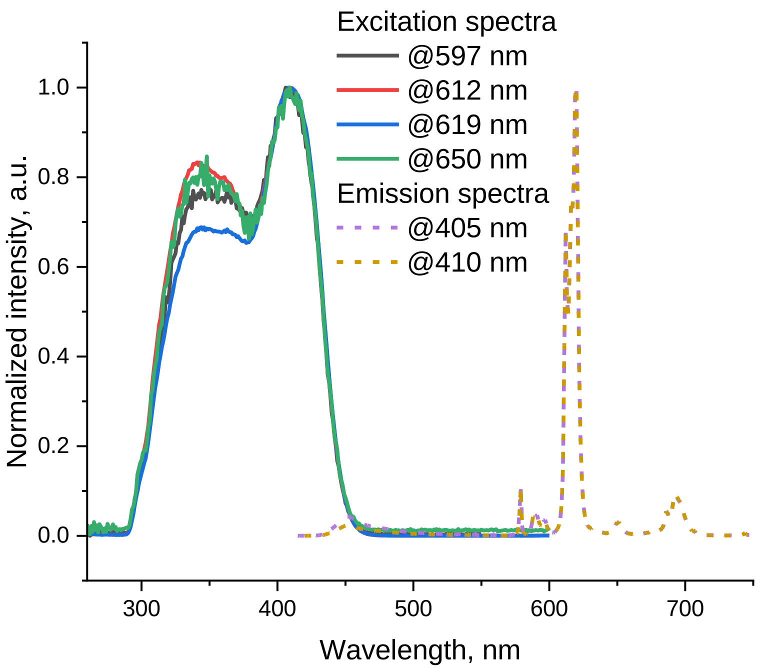

Figure S17. Excitation (solid lines) and emission (dashed lines) spectra of complex **Eu2** in monomers taken with the same amount as for polymerization, 293K.

Eu3

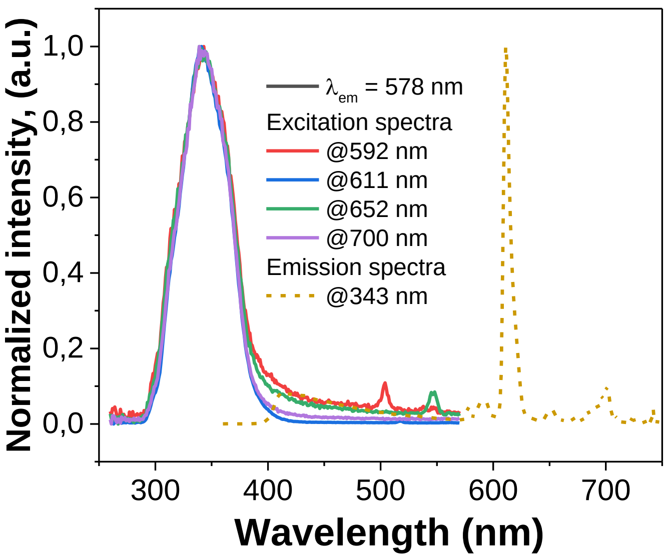

Figure S18. Excitation (solid lines) and emission (dashed lines) spectra of complex **Eu3** in monomers taken with the same amount as for polymerization, 293K.

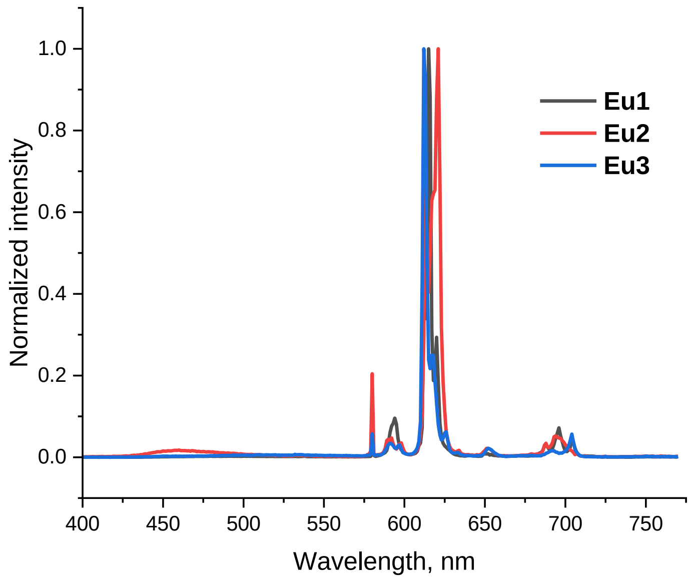

Figure S19. Two-photon emission spectra of europium complexes in dichloromethane solution using 800 nm excitation wavelength, 293K.

The temperature dependence of the excited state lifetime (τ) was approximated according to the equation:

$$\tau(T) = \frac{1}{k_r + A \cdot e^{-\frac{\Delta E}{k_B T}}} \sim A' \cdot e^{\frac{\Delta E}{k_B T}} \qquad \textbf{Equation S1}$$

where

k_r is the rate of radiative transition;

k_{nr} is the rate of nonradiative transition;

A is a constant weakly dependent on temperature;

k_B is the Boltzmann constant;

T is temperature.

Synthesis of Eu-containing nanoparticles

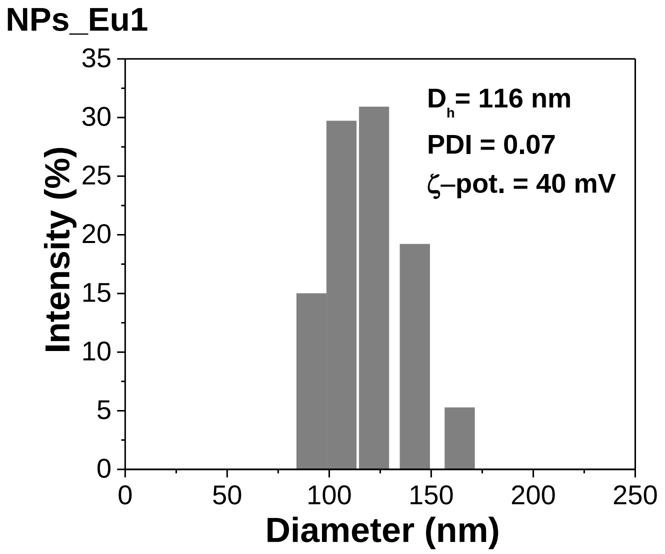

Figure S202. Main characteristics of **NPs_Eu1**. D_h – hydrodynamic diameter, PDI – polydispersity index, ζ-pot. - ζ-potential.

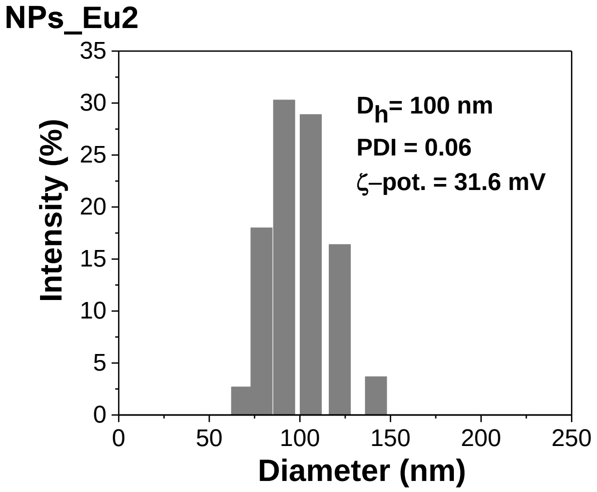

Figure S213. Main characteristics of **NPs_Eu2**. D_h – hydrodynamic diameter, PDI – polydispersity index, ζ-pot. - ζ-potential.

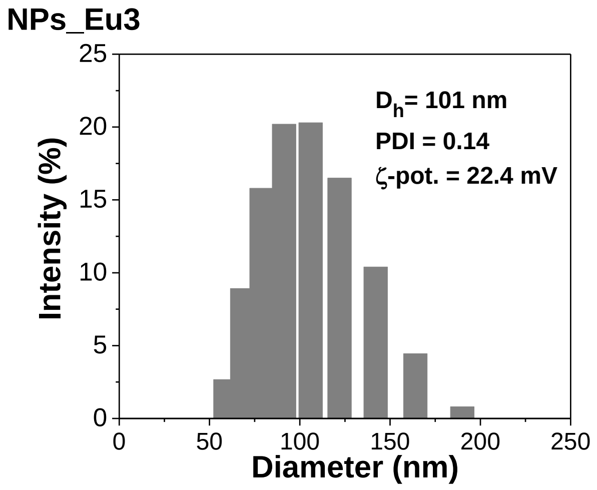

Figure S224. Main characteristics of **NPs_Eu3**. D_h – hydrodynamic diameter, PDI – polydispersity index, ζ-pot. - ζ-potential.

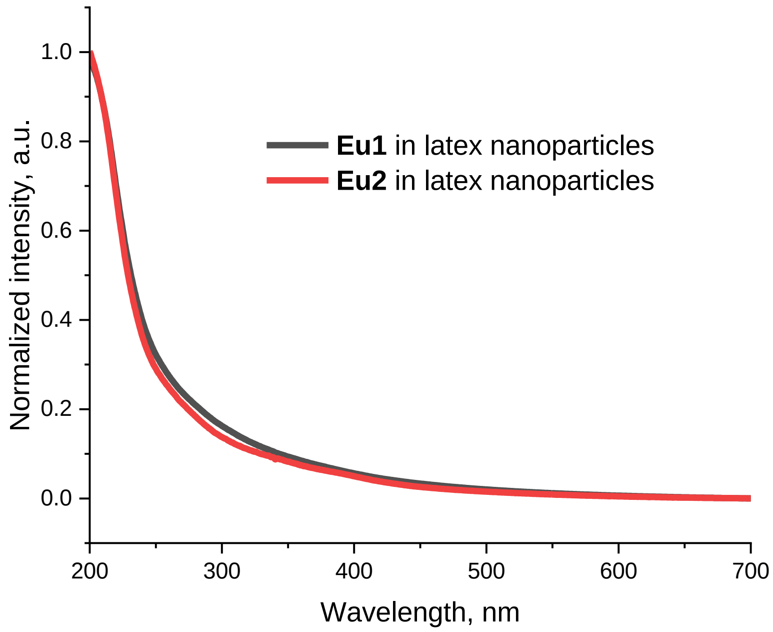

Figure S23. Absorption spectra of latex nanoparticles in water dispersion, 293K.

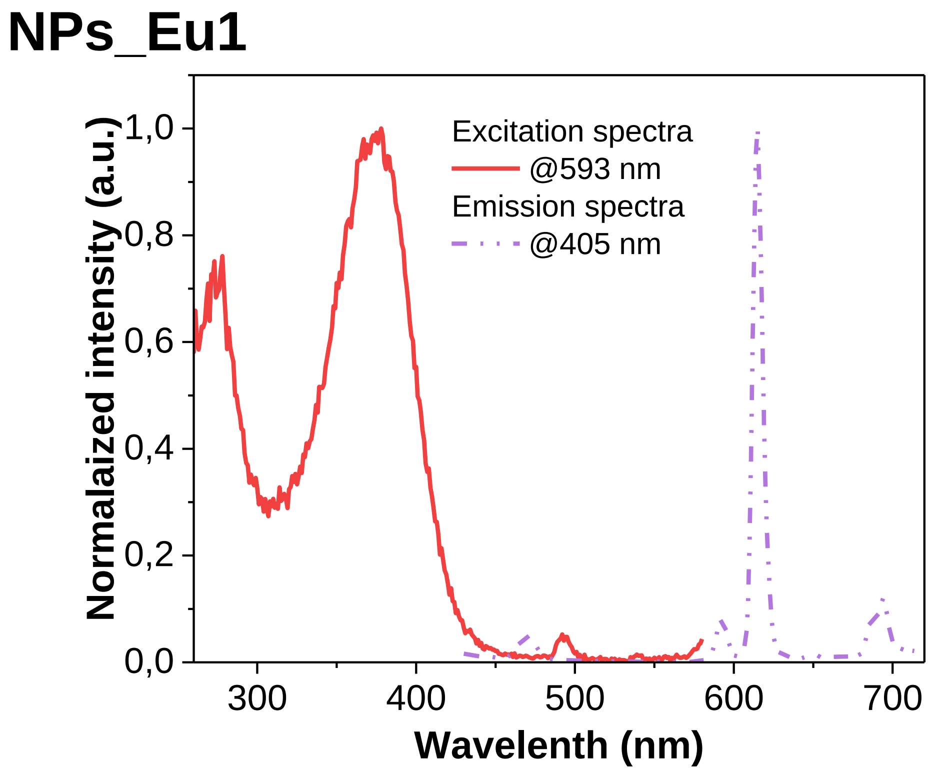

Figure S24. Excitation (solid lines) and emission (dashed lines) spectra of nanoparticles with **NPs_Eu1** in water dispersion, 293K.

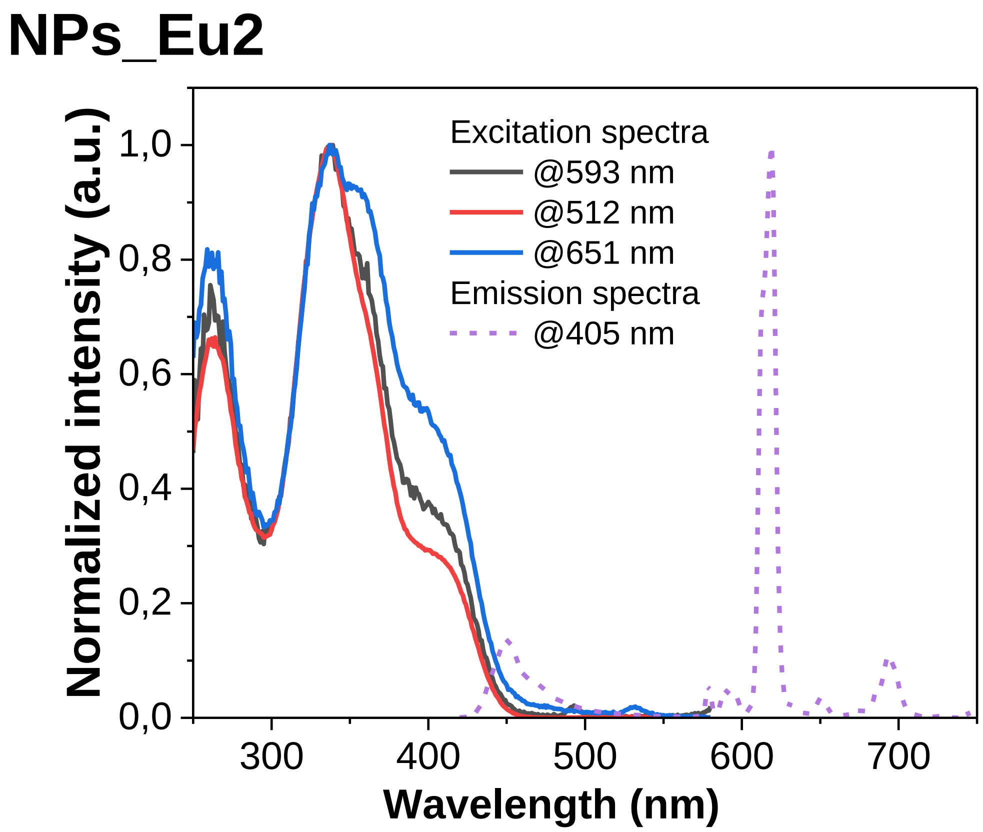

Figure S25. Excitation (solid lines) and emission (dashed lines) spectra of nanoparticles with **NPs_Eu2** in water dispersion, 293K.

NPs_Eu3

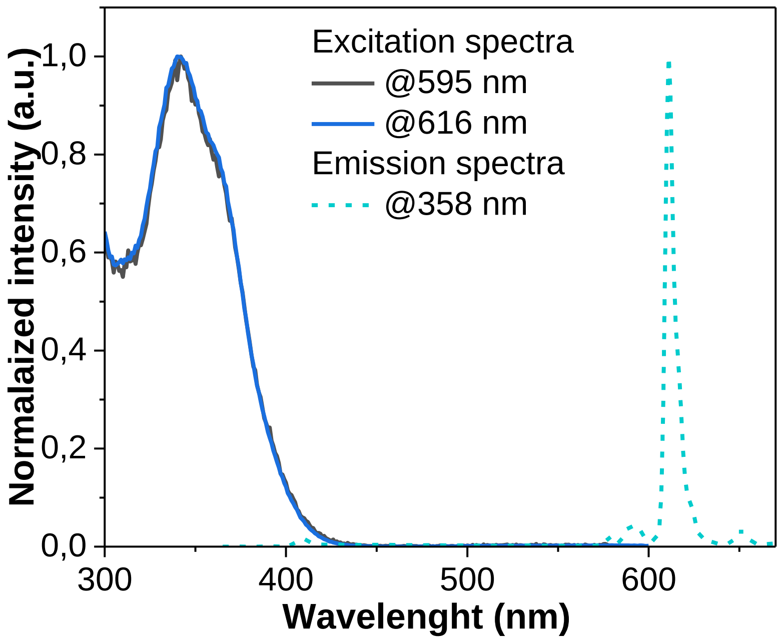

Figure S26. Excitation (solid lines) and emission (dashed lines) spectra of nanoparticles with **NPs_Eu3** in water dispersion, 293K.

Determination of the number of lanthanide complexes and triphenylphosphine per particle

The number of latex particles per milliliter is given by the following equation [bangslab.com URL https://www.bangslabs.com/sites/default/files/imce/docs/TechNote%20206%20Web.pdf (achieved on 04.10.2022)]:

$$N_p = \frac{6 \cdot 10^{10} \cdot S \cdot \rho_L}{\pi \cdot \rho_S \cdot d^3}, \qquad \textbf{Equation S2}$$

where

N_p is number of latex nanoparticles per milliliter;

S is weight % solids (for 10% solids suspension S=10);

ρ_L is density of nanoparticle suspension (g/mL);

$$\rho_L = \frac{100 \cdot \rho_S}{S \cdot (1 - \rho_S) + (100 \cdot \rho_S)};$$

ρ_L is density of solid nanoparticle (g/cm^3);

d is mean diameter (µm).

The number of europium complexes (or phosphonium salt) per particle was then calculated according to:

$$N_{Eu} = \frac{n_{Eu} \cdot N_A}{N_p} \text{ (or } N_P = \frac{n_P \cdot N_A}{N_p}\text{)},$$

where

N_p is number of latex nanoparticles per milliliter;

n_{Eu} and n_P is the quantity of incorporated europium complexes or phosphonium salt)(mol) determined by ICPOES;

N_A is the Avogadro number (mol^{-1}).

The lifetime sensor characteristics

The sensors characteristics have been calculated in accordance with the literature guidelines [Miroslav D. Dramićanin, *J. Appl. Phys.* 128, 040902 (2020); doi: 10.1063/5.0014825].

Temperature sensitivity (S_T)

$$S_T = \frac{d\tau}{dT}$$ **Equation S3**

Dividing this magnitude by the indicator value (τ_{in}), which is the minimum in the range under study, we obtain the relative sensitivity (S_r):

$$S_r = \frac{d\tau/dT}{\tau_{in}}$$ **Equation S4**

Reproducibility (*R*) was determined by measuring ten cycles with a change in the signal value from τ to τ_i:

$$R = 1 - \frac{\tau-\tau_i}{\tau}$$ **Equation S5**

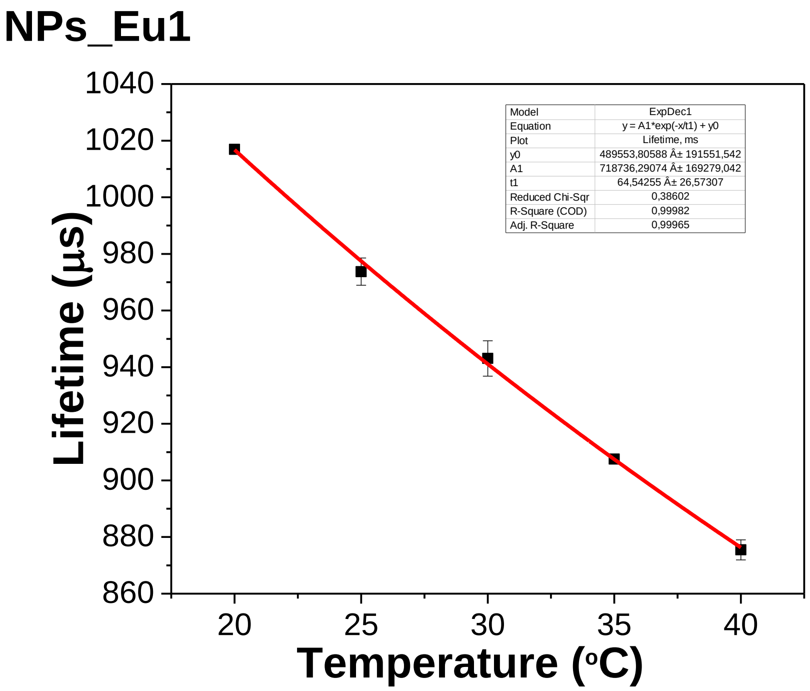

Figure S27. Correlation between lifetimes of excited state and temperature for **NPs_Eu1** in water dispersion.

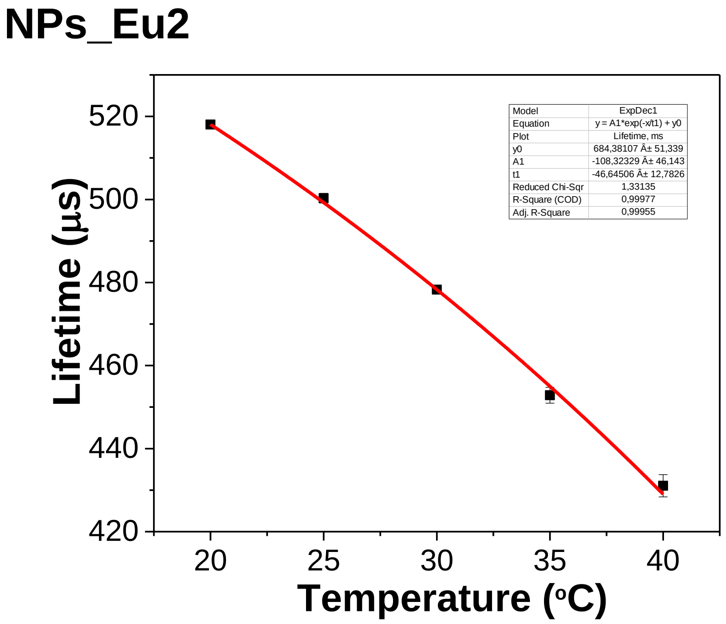

Figure S285. Correlation between lifetimes of excited state and temperature for **NPs_Eu2** in water dispersion.

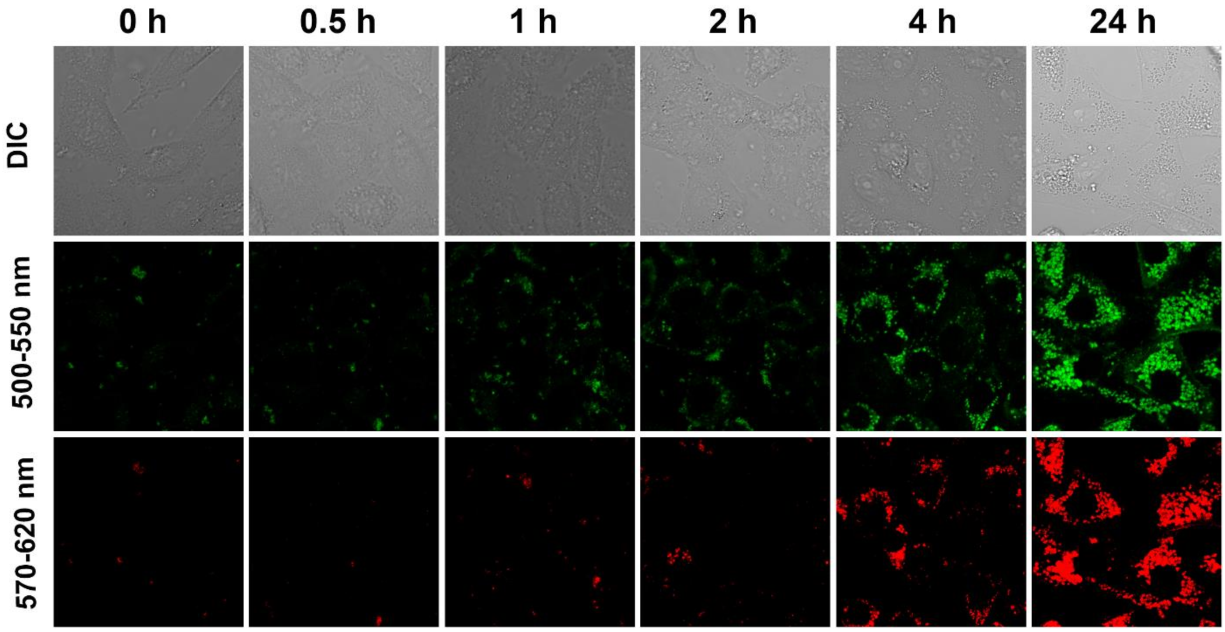

Figure S29. Dynamics of **NPs_Eu2** (0.0195 wt%,) accumulation by CHO-K1 cells during long-term incubation carried out in DMEM-F12 supplemented with 10% FBS. Confocal images: DIC image (top); green channel 500-550 nm (middle); red channel 570-620 nm (bottom). Scale bar 20 µm.

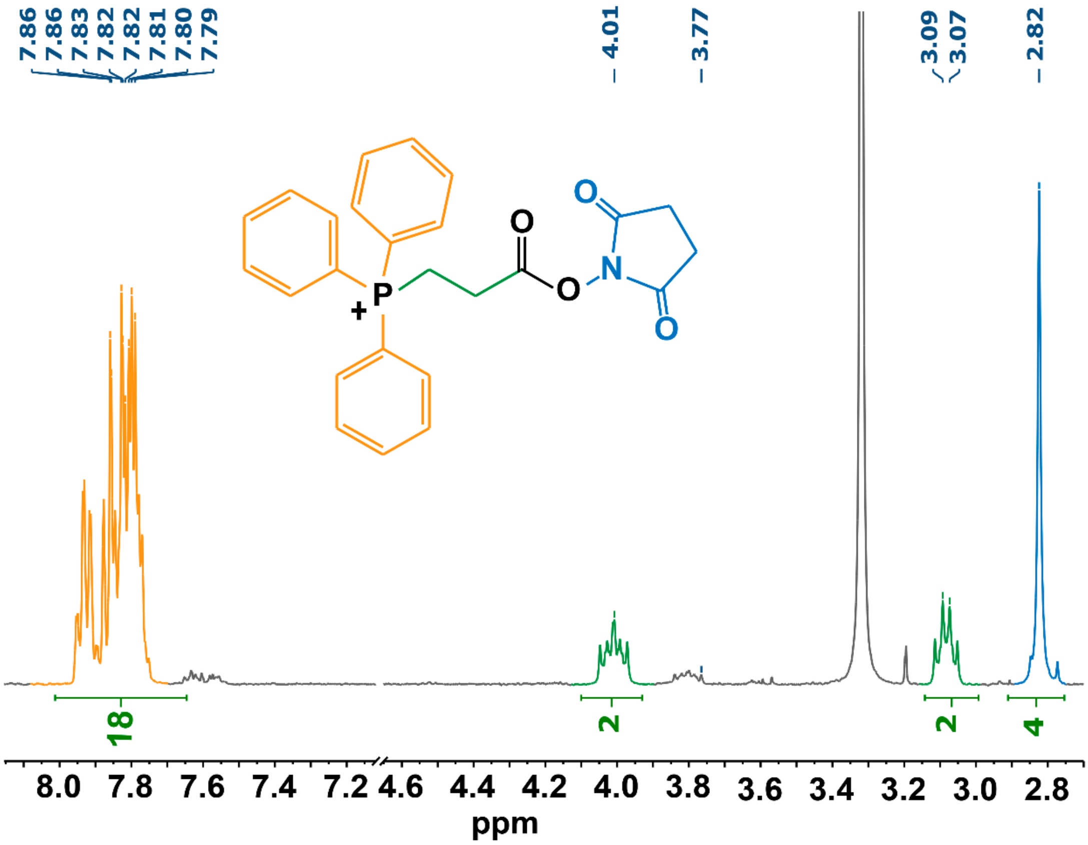

(A)

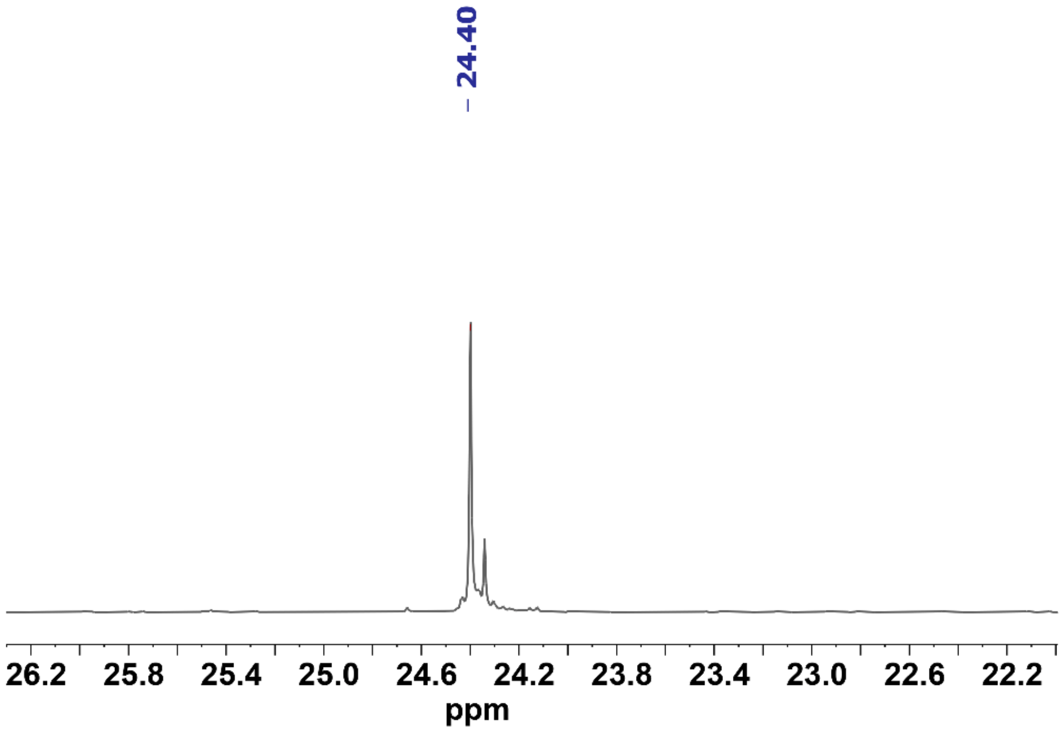

(B)

Figure S30. The NMR spectra of **TPP-NHS ester** (A) ^{1}H, (B) ^{31}P.

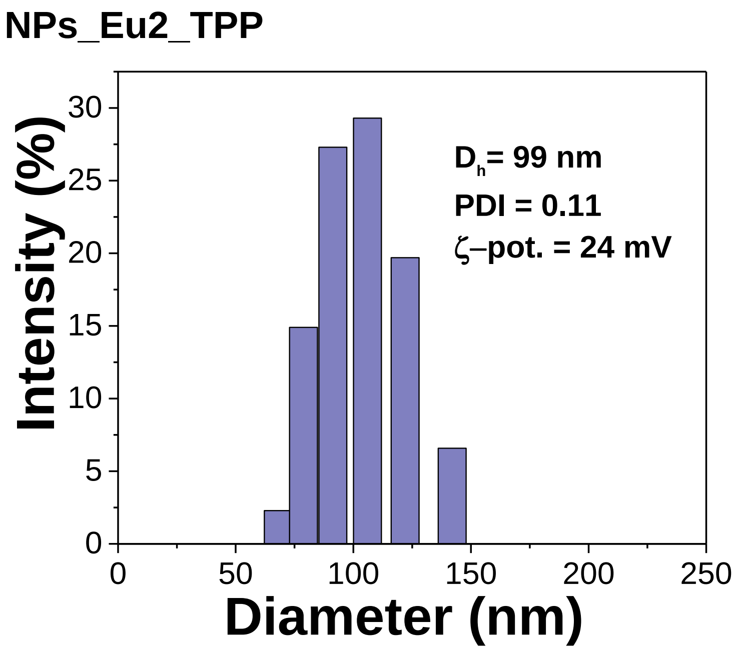

Figure S316. Main characteristics of **NPs_Eu2_TPP**. D_h – hydrodynamic diameter, PDI – polydispersity index, ζ-pot. - ζ-potential.

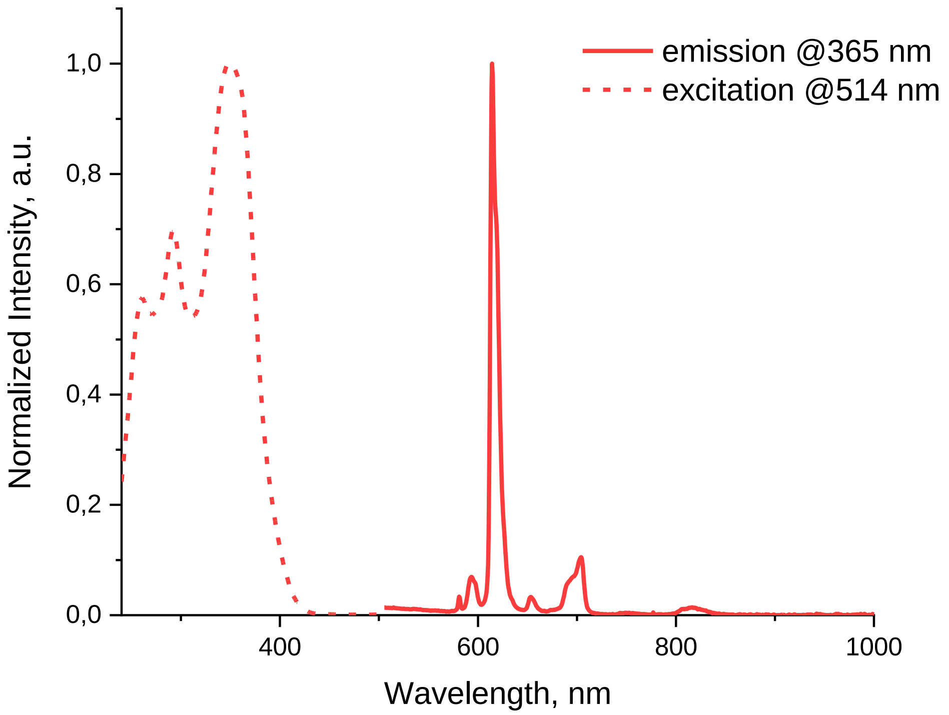

Figure S32. Excitation (dashed line) and emission (solid line) spectra of nanoparticles with **NPs_Eu2_TPP** in water dispersion, 293K.

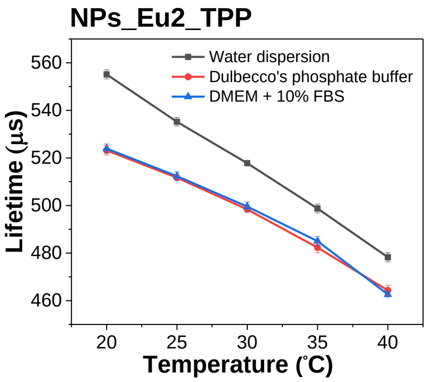

(a)

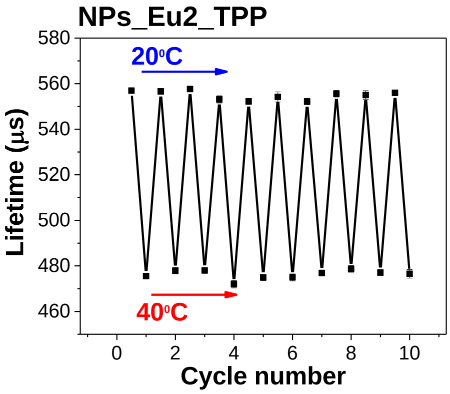

(b)

Figure S33. (a) Temperature sensitivity of NPs_Eu2_TPP lifetime in different media: water, Dulbecco's buffer and model physiological medium (DMEM+10%FBS). Interpolation of experimental data has been done using Equation 1; (b) Cyclic lifetime measurements for NPs_Eu2 between 20 and 40°C for NPs_Eu2 in water and DMEM + 10% FBS.

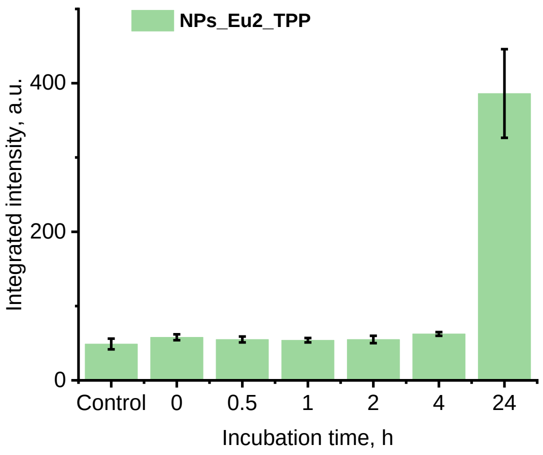

(a)

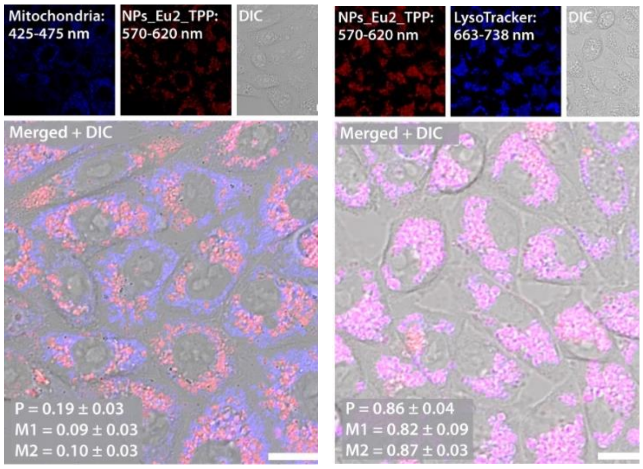

(b) (c)

Figure S34. (a) The dependence of emission intracellular intensity vs incubation time of CHO-K1 with NPs_Eu2_TPP (b) Subcellular distribution of BioTracker 405 Blue Mitochondria Dye (blue color) and NPs_Eu2_TPP (red color) in CHO-K1 cells; (c) Subcellular distribution of LysoTracker Deep Red (blue color), and NPs_Eu2_TPP (red color) in CHO-K1 cells. Pearson's (P) and Manders' overlap coefficients (M1 – fraction of tracker signal that overlaps conjugate signal, M2 – fraction of conjugate signal that overlaps tracker signal) are presented as mean ± standard deviation calculated for 50 cells. Scale bar 20 µm.

Table S1. Temperature-dependent emission lifetimes of NPs_Eu2 and NPs_Eu2_TPP in media of various nature.

Temperature, ˚C	Lifetime, μs									
	NPs_Eu2							NPs_Eu2_TPP		
	Water	Dulbecco`s phosphate buffer (pH=7.4)	DMEM +10% FBS	Water/glycerin mixture, wt.%		Citrate-phosphate buffer		Water	Dulbecco`s phosphate buffer (pH=7.4)	DMEM +10% FBS
				90:10	50:50	pH=4.7	pH=7.2			
20	518	496	493	533	524	530	525	555	523	524
25	500	481	478	-	-	-	-	535	512	512
30	478	462	458	-	-	-	-	518	498	499
35	453	440	436	-	-	-	-	498	482	485
40	431	420	410	447	449	437	435	478	464	463

Table S2. A comparison of ***Polymer NPs-Eu2*** nanothermometer characteristics with those of published luminescent lifetime probes.

Probe	Measurment mode	Temperature range, °C	Excitation/Emission wavelengths, nm	Lifetime Range	Sensitivity, %/K	Reference
Ultra-Long-Lived Luminescent Nanocapsule loaded by $(PdPc(OBu)_8$ and PCU	Fluorescence Lifetime	20-40	375/444, 475, 508	0.3-1.52 s	7.5	[38]
HPS/Butter/ DSPE-PEG-Biotin Nanorod based on AIE material	Fluorescence Lifetime	20-60	375, 405sh/490	1-2.5 ns	6.3	[39]
Gold Nanoclusters (AuNCs)	Fluorescence Lifetime	10-50	360, 402sh/603	2.8-7.9 µs	2.8	[40]
ER thermo yellow	Fluorescence Lifetime	23-40	560/600	2.2-2.6 ns	1.04	[41]
YAG:Ce NPs	Fluorescence Lifetime	7-77	346/560	18.5-26.5 ns	0.2	[42]
C70 in PtBMA	Fluorescence Lifetime	20-90	470/700	17.5-27.0 ms	0.5	[43]
Eu-DT in BTD-PMMA	Phosphorescence Lifetime	25-45	400/616	145-350 µs	2.2	[44]
Gold Nanoclusters	Fluorescence Lifetime	15-45	580/710	460-600 ns	0.5	[45]
Fluorescent polymer	Fluorescence Lifetime	28-40	456/565	4.5-7.5 ns	0.6	[46]
First generation of luminescent polymer NPs with Eu complex	Phosphorescence Lifetime	25-45	335/614	509-580 µs	0.84	[31]
Polymer NPs-Eu2	Phosphorescence Lifetime	20-40	405/614	430-520 µs	1.3	This work

References

38. Su, X.; Wen, Y.; Yuan, W.; Xu, M.; Liu, Q.; Huang, C.; Li, F. Lifetime-Based Nanothermometry *in Vivo* with Ultra-Long-Lived Luminescence. *Chemical Communications* **2020**, *56*, 10694–10697, doi:10.1039/D0CC04459H.

39. Gao, H.; Kam, C.; Chou, T.Y.; Wu, M.-Y.; Zhao, X.; Chen, S. A Simple yet Effective AIE-Based Fluorescent Nano-Thermometer for Temperature Mapping in Living Cells Using Fluorescence Lifetime Imaging Microscopy. *Nanoscale Horizons* **2020**, *5*, 488–494, doi:10.1039/C9NH00693A.

40. Wang, Y.; Liang, S.; Mei, M.; Zhao, Q.; She, G.; Shi, W.; Mu, L. Sensitive and Stable Thermometer Based on the Long Fluorescence Lifetime of Au Nanoclusters for Mitochondria. *Analytical Chemistry* **2021**, *93*, 15072–15079, doi:10.1021/acs.analchem.1c03092.

41. Itoh, H.; Arai, S.; Sudhaharan, T.; Lee, S.-C.; Chang, Y.-T.; Ishiwata, S.; Suzuki, M.; Lane, E.B. Direct Organelle Thermometry with Fluorescence Lifetime Imaging Microscopy in Single Myotubes. *Chemical Communications* **2016**, *52*, 4458–4461, doi:10.1039/C5CC09943A.

42. Allison, S.W.; Gillies, G.T.; Rondinone, A.J.; Cates, M.R. Nanoscale Thermometry via the Fluorescence of YAG:Ce Phosphor Particles: Measurements from 7 to 77 C. *Nanotechnology* **2003**, *14*, 859–863, doi:10.1088/0957-4484/14/8/304.

43. Baleizão, C.; Nagl, S.; Borisov, S.M.; Schäferling, M.; Wolfbeis, O.S.; Berberan-Santos, M.N. An Optical Thermometer Based on the Delayed Fluorescence of C70. *Chemistry - A European Journal* **2007**, *13*, 3643–3651, doi:10.1002/chem.200601580.

44. Peng, H.; Stich, M.I.J.; Yu, J.; Sun, L.; Fischer, L.H.; Wolfbeis, O.S. Luminescent Europium(III) Nanoparticles for Sensing and Imaging of Temperature in the Physiological Range. *Advanced Materials* **2010**, *22*, 716–719, doi:10.1002/adma.200901614.

45. Shang, L.; Stockmar, F.; Azadfar, N.; Nienhaus, G.U. Intracellular Thermometry by Using Fluorescent Gold Nanoclusters. *Angewandte Chemie International Edition* **2013**, *52*, 11154–11157, doi:10.1002/anie.201306366.

46. Okabe, K.; Inada, N.; Gota, C.; Harada, Y.; Funatsu, T.; Uchiyama, S. Intracellular Temperature Mapping with a Fluorescent Polymeric Thermometer and Fluorescence Lifetime Imaging Microscopy. *Nat Commun* **2012**, *3*, 705, doi:10.1038/ncomms1714.

31. Shakirova, J.R.; Shevchenko, N.N.; Baigildin, V.A.; Chelushkin, P.S.; Khlebnikov, A.F.; Tomashenko, O.A.; Solomatina, A.I.; Starova, G.L.; Tunik, S.P. Eu-Based Phosphorescence Lifetime Polymer Nanothermometer: A Nanoemulsion Polymerization Approach to Eliminate Quenching of Eu Emission in Aqueous Media. *ACS Appl Polym Mater* **2020**, *2*, 537–547, doi:10.1021/acsapm.9b00952
